# Supplementary material for: Splice-Junction-Based Mapping of Alternative Isoforms in the Human Proteome
Source: Cell Rep. Author manuscript; Available in PMC 2020 Jan 15. (PMC6961840; doi:10.1016/j.celrep.2019.11.026)
Supplement: 3 [file NIHMS1546469-supplement-3.zip › DF2/PXD006675/LeftAtrium-285-Q9H2G2-KEEQEFVQK.pdf]

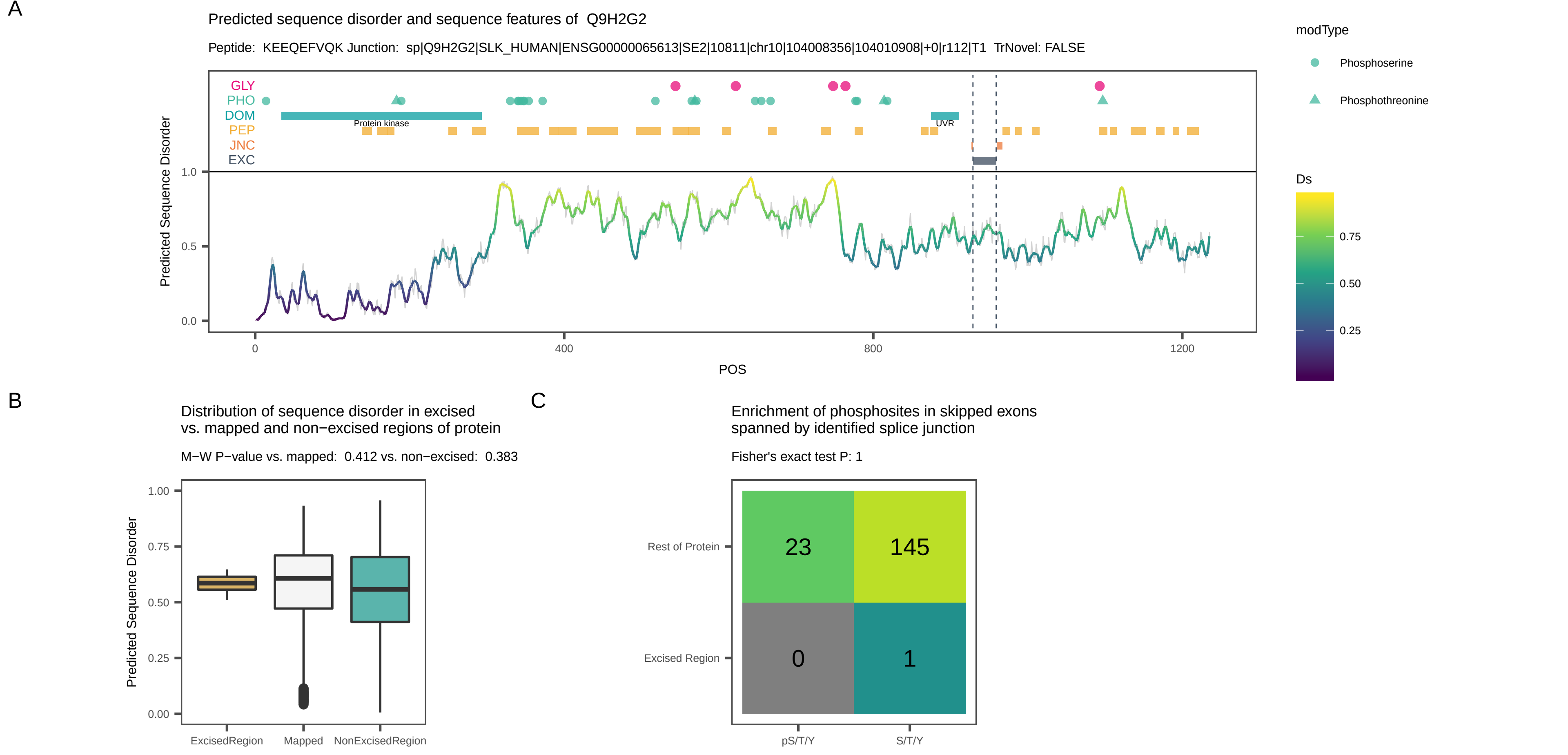
A
Predicted sequence disorder and sequence features of Q9H2G2
Peptide: KEEQEFVQK Junction: sp|Q9H2G2|SLK_HUMAN|ENSG00000065613|SE2|10811|chr10|104008356|104010908|+0|r112|T1 TrNovel: FALSE
GLY
PHO
DOM
PEP
JNC
EXC
Protein kinase
UVR
Predicted Sequence Disorder
1.0
0.5
0.0
0
400
800
1200
POS
modType
Phosphoserine
Phosphothreonine
Ds
0.75
0.50
0.25
B
Distribution of sequence disorder in excised vs. mapped and non−excised regions of protein
M−W P−value vs. mapped: 0.412 vs. non−excised: 0.383
Predicted Sequence Disorder
1.00
0.75
0.50
0.25
0.00
ExcisedRegion
Mapped
NonExcisedRegion
C
Enrichment of phosphosites in skipped exons spanned by identified splice junction
Fisher's exact test P: 1
Rest of Protein
Excised Region
23
145
0
1
pS/T/Y
S/T/Y
